# Pleural and Pericardial Effusion With COVID-19 and Systemic Lupus Erythematosus and Its Recurrence: A Case Study

**DOI:** 10.7759/cureus.37988

**Published:** 2023-04-22

**Authors:** Connor Yost, Darin Vercillo, Abdulelah Abuqare, Mckenna b Yost, Avery N Love

**Affiliations:** 1 Internal Medicine, A.T. Still University-School of Osteopathic Medicine in Arizona (ATSU-SOMA), Mesa, USA; 2 Internal Medicine, Davis Hospital and Medical Center, Layton, USA

**Keywords:** systemic lupus erythematosis, pericardiocentesis, pericarditis, coronavirus, cardiac tamponade

## Abstract

As a chronic autoimmune disease, systemic lupus erythematosus (SLE) primarily affects young women and does not discriminate against any particular organs. In December 2019, coronavirus disease 2019 (COVID-19) spread worldwide, with many speculations of cardiac involvement in the pathogenesis of infection. Moreover, in cases where cardiac symptoms were described, they consisted solely of chest pain or a general deterioration in health if the patient presented with pleural effusion or pericardial effusion.

Our patient, a 25-year-old Hispanic woman, initially complained of chest pain, cough, and shortness of breath. After being admitted, she noticed growing dyspnea and mild discomfort on the right side of her chest. The patient had both SLE and COVID-19 and had developed pleural and pericardial effusions. After two days in culture, nothing had grown from the fluid samples. In addition, measures of brain natriuretic peptide and total creatine kinase fell within the normal range. Considering the investigational findings, pericardiocentesis was performed. After the procedure, the patient's condition improved, and she was discharged. The patient continued taking CellCept® 1,500 mg and Plaquenil 200 mg and started taking colchicine. Her daily prednisone dose was increased to 40 milligrams. She felt well initially; however, after two weeks of follow-up, the pericardial effusion recurred, and pericardiocentesis was performed again. The patient was discharged in stable condition after a two-day hospital stay.

After treatment of both initial and recurrent effusions, the patient’s cardiac symptoms were resolved, and blood pressure became stable. We hypothesize that there may be other unreported cases of COVID-19-related viral pericarditis, pericardial effusion, and pericardial tamponade that could be caused by a combination of COVID-19 and a pre-existing condition, mainly autoimmune disorders. Due to the lack of clarity surrounding typical COVID-19 manifestations, it is crucial to record all cases of this unique illness and analyze any increased incidence of pericarditis, pericardial effusion, and pericardial tamponade in the public.

## Introduction

Autoimmune disorders have been a common cause of cardiac symptoms in the public. One in particular that can lead to pericardial effusions and potentially cardiac tamponade is systemic lupus erythematosus (SLE). SLE occurs in a population at a rate of 0.3-1.5% per 1,000 people [1].

In December 2019, coronavirus disease 2019 (COVID-19) began spreading globally. Much is unknown about this novel virus, but viral knowledge and new clinical manifestations/presentations are emerging daily. Acute cardiac injury, a significant risk of thrombosis, stroke, pulmonary embolism, and acute coronary syndrome, are all associated with COVID-19, and evidence has been found that this virus may greatly impact the cardiovascular system [2]. Nonetheless, pericardial effusion has received comparatively little attention. Therefore, COVID-19 patients with chest discomfort should be evaluated for pericardial effusion, pleural effusion, and cardiac tamponade [3].

Chest pain or a general decline in health was the only symptom reported in the small number of cases of pericardial effusion and pleural effusion [3]. Pericardial effusion is increased fluid around the heart, and this process can lead to a rise in pericardial cavity pressure that may limit ventricular filling and lower cardiac output, a condition known as cardiac tamponade [4]. Pleural effusion is fluid between the pleural layers of the lungs, which leads to impaired expansion. Pericardial and pleural effusions are either transudative or exudative. Transudative effusion is caused by heart failure, post-open-heart surgery, post-myocardial infarction, and infection while exudative effusion is caused by pulmonary bacterial pneumonia, tuberculosis, cancer, and inflammatory disorders like pancreatitis, lupus, rheumatoid arthritis, and post-cardiac injury syndrome (mainly pericardial). Patients with COVID-19 and SLE may experience pericardial effusion consequently [5,6]. Mild, bilateral pleural effusion is also common in people with COVID and SLE [5,6]. Due to the occurrence rates in both pathologies, patients with SLE and COVID should be evaluated with a broad differential diagnosis and early ultrasonography guidance [3].

Moreover, it has been shown that it is beneficial to drain the pericardial effusion first in individuals who present with tamponade, then the pleural effusion [7]. Most cases of pericardial effusion and pleural effusion can be diagnosed by analyzing the patient's medical history, physical exam, chest X-ray, and a diagnostic thoracentesis for pleural effusions [8]. In many cases, both pleural effusion and pericardial effusion can resolve on their own. In severe cases, therapeutic thoracentesis is required for pleural effusions, and pericardiocentesis is required for pericardial effusions [8]. After severe pericardial effusion and pleural effusion are confirmed, pericardiocentesis should be performed immediately because it is a life-saving intervention for acute cardiac tamponade (with unstable hemodynamics). The Mayo Clinic experience found that echocardiography-guided pericardiocentesis was a straightforward, safe, and very successful treatment for large postoperative pericardial effusions [9].

Sometimes, even after a thorough investigation, the root cause of effusion remains unknown. A long list of conditions might cause pericardial and pleural effusion to persist without a diagnosis [10]. This report describes a 25-year-old woman who presented with pericardial and pleural effusion and was diagnosed with COVID-19 infection and SLE.

## Case presentation

The patient in the intensive care unit (ICU) was a 25-year-old Hispanic woman with right pleural effusion and pericardial effusion. Increasing dyspnea and slight right lateral chest discomfort had been reported. The patient had COVID-19 in addition to a history of SLE for seven years. Upon admission to the ICU, the patient had normal heart sounds except for a slight low-tone rub, regular rate, regular rhythm, no edema, and no jugular vein distention. Additionally, left-sided crackles were heard during auscultation.

**Figure 1:**
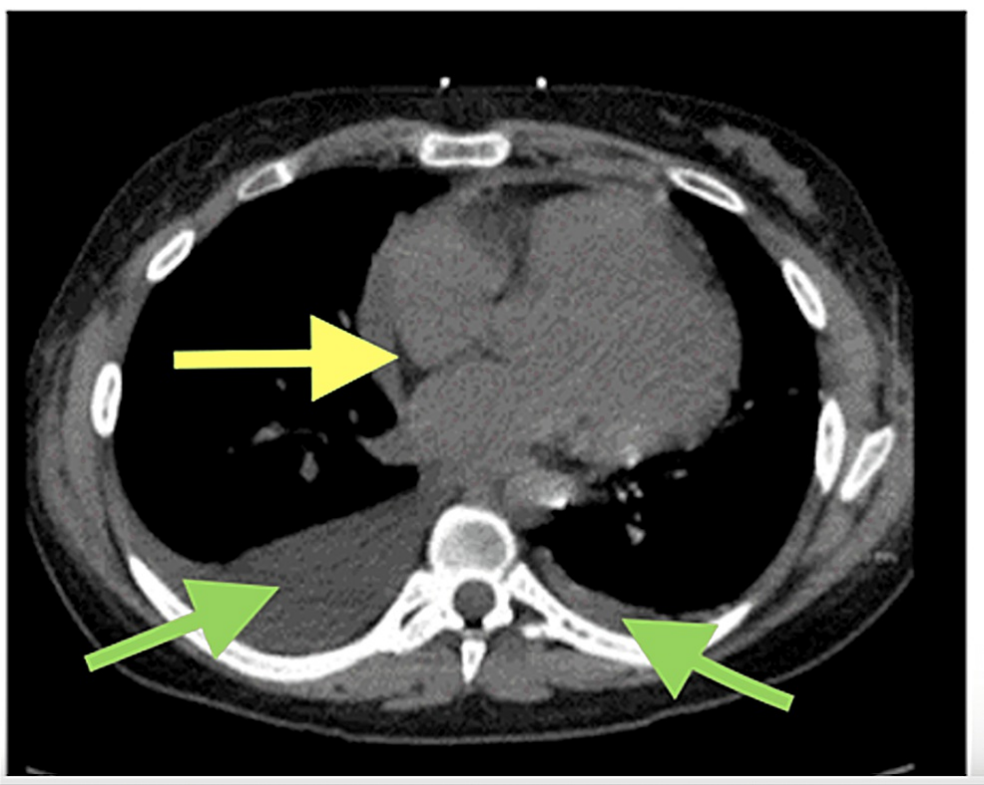
CT without contrast after transverse pericardiocentesis Yellow arrows indicate the pericardial space; Green arrows indicated pleural effusions

A CT was performed and showed a likely pericardial effusion and pleural effusion, as seen in Figure 1. After careful consideration, a thoracentesis was performed while waiting on a pericardiocentesis to see if the effusion would decrease in size on its own. An ultrasound-guided thoracentesis was performed, yielding 660 ml of clear, yellow fluid. Post-ultrasound, no pleural effusion was found. Lab tests showed a lactate dehydrogenase (LDH) level of 139. In addition, right and left peripheral blood cultures were also done. No growth was found after two days of culture. On the patient’s fourth day in the hospital, she became slightly hypoxic and she underwent ultrasound-guided right-sided thoracentesis again for diagnostic and therapeutic purposes, which she tolerated well. The removal of fluid again led to the resolution of recurrent hypoxia and gradually alleviated her shortness of breath over the six hours following the procedure. A repeat CT scan without contrast was performed, which showed a decrease in her pericardial effusion and pleural effusions.

We maintained her home medications of CellCept 1,500 mg, Plaquenil 200 mg, and Methotrexate 20 mg, and added colchicine while increasing her prednisone dosage to 40 mg from 15 mg daily. The patient tolerated the medication well and reported a decrease in chest pain and shortness of breath. She had chronic anemia that did not improve while she was hospitalized. The patient’s kidney function remained normal. She was discharged home in stable condition and instructed to follow up with cardiology, as well as her rheumatologist over the next two weeks to monitor the tapering of her steroids. She was instructed to taper her prednisone by 10 mg every week after two weeks of treatment. She was also provided with symptomatic medication, including Tessalon Perles and tramadol as needed for pain. The patient voiced understanding of the plan of care and agreed to proceed. She was instructed to avoid strenuous activity and was able to return to work the following week. Lab values for admission and discharge are found in Table 1.

**Table 1:** Laboratory test results on admission and discharge MCV: mean corpuscular volume; MCHC: mean corpuscular hemoglobin concentration; RDW: red cell distribution width; GFR: glomerular filtration rate

Test	Admission	Discharge	Reference range
RBC (L)	3.89	3.97	4.20-5.50
WBC (L)	5.0	2.8	4.5 to 11.0 × 10^9^/L
Hgb (g/dL)	8.4	-	12-16
MCV (L)	74.0	70.3	80-95
MCHC (g/dL)	29.2	31.1	31-36
RDW (%)	21.6	-	11.5-14.5
Plt count (x10^3^/uL)	427	327	147-425
Neutrophils (%)	79.3	55.2	40-75
Lymphocytes (%)	17.8	29.4	20-40
Estimated GFR (ml)	79	128	>=90 ml/min/1.73 m^2^
Calcium (mg/dL)	6.8	7.1	8.7-10.4

Recurrence of pericardial and pleural effusion

At the time of her discharge from the hospital, she had improved significantly but still experienced dyspnea on exertion. Three weeks later, she was admitted again for severe diffuse pleuritic chest pain, which was rated as 10/10 in terms of severity, but she did not have a fever. Her COVID test on readmission was negative. Her dose of prednisone had been decreased during the previous work from 40 mg to 30 mg daily. Various lab tests, an electrocardiogram (EKG), and a computed tomography angiography (CTA) chest with contrast were suggested emergently.

**Figure 2:**
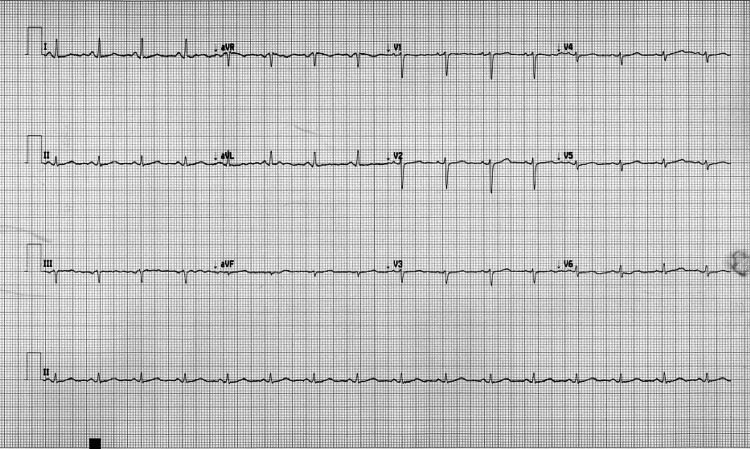
EKG before follow-up

**Figure 3:**
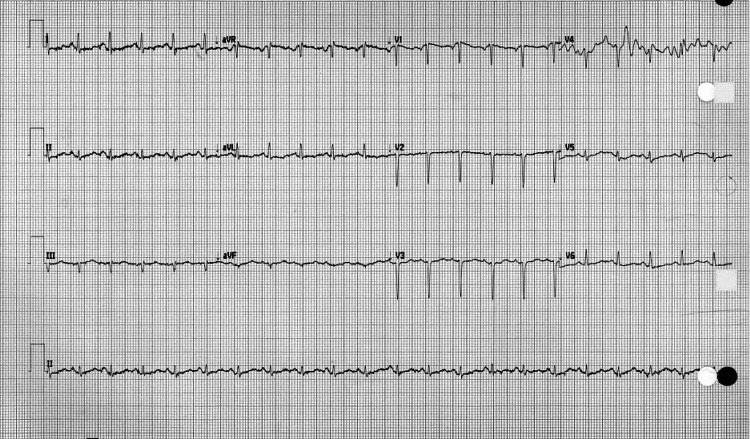
Follow-up EKG shows sinus tachycardia

The EKG showed a sinus tachycardia rate of 129 bpm and no ectopy. Despite certain limitations in interpretation brought on by artifacts, no acute ischemic alteration is readily apparent. When comparing this EKG to a previous one, no major differences were found, which was intriguing given the presence of cardiac tamponade (Figures 2, 3).

**Figure 4:**
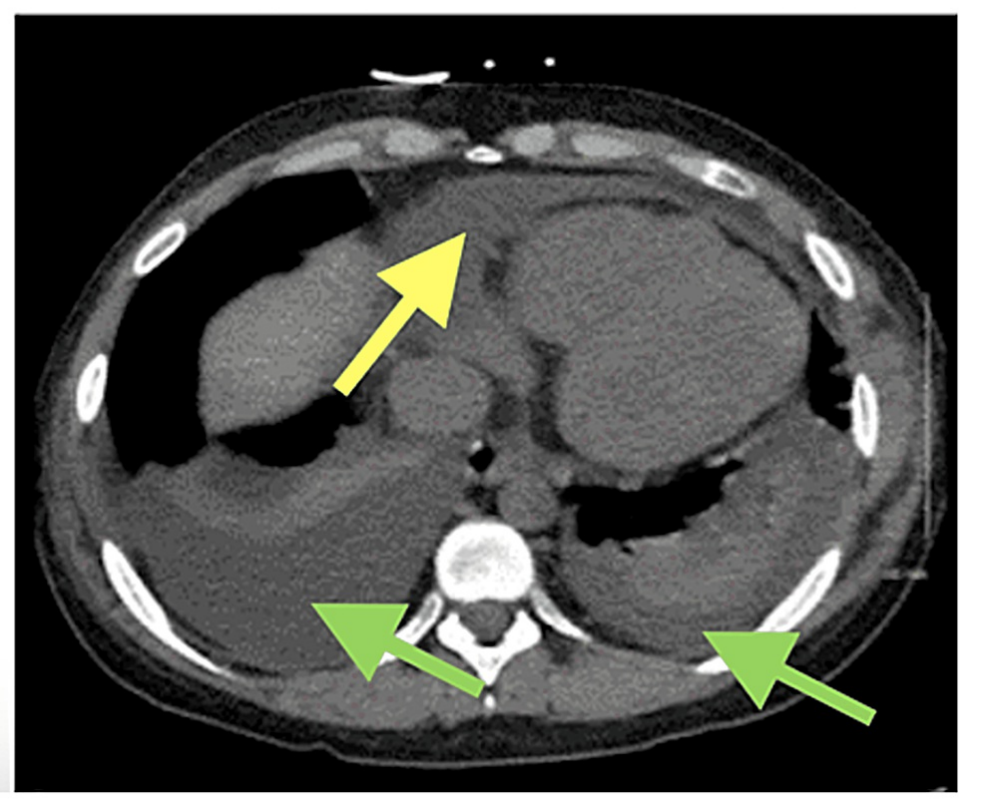
CT scan without contrast shows the recurrence of cardiac tamponade three weeks after Yellow arrows indicate pericardial effusion; Green arrows indicate pleural effusions

A CT scan without contrast (Figure 4) was performed and was compared to her previous visit and post-pericardiocentesis CT. The CT scan revealed recurring pericardial and pleural effusions. An echocardiogram showed a significant effusion and early signs of tamponade, including a collapsed right atrium during systole; however, the patient was not hypotensive. Due to her previous diagnosis of cardiac tamponade, it was assumed that she would again experience tamponade if the pericardial effusion was left untreated. Pericardiocentesis was again suggested, and 350 ml of serous fluid was successfully extracted.

A drain was left in the pericardium for 24 hours. On day 4 of her second hospital stay, a repeat echocardiogram showed a reduction of the pericardial effusion, and the patient was again discharged. The pain was treated with intravenous Toradol and Percocet. She was restarted on colchicine at an increased dose of .6 mg, which we found to be effective in treating her pain as well. The patient was instructed to resume home medication and increase the prednisone dosage to 40 mg for three weeks with a one-week follow-up. It is also to be noted that discharge lab work showed leukopenia when the WBC count had been within the normal range upon admission. Due to the increase in steroid dosage, we would expect her WBC count to increase, but during her hospital treatment, her WBC count decreased significantly (Table 2). This followed the same trend as her previous hospital visit, though the decrease was much more substantial during the second hospital visit.

**Table 2:** Laboratory test results on admission and discharge (recurrence) after two weeks MCV: mean corpuscular volume; MCHC: mean corpuscular hemoglobin concentration; RDW: red cell distribution width; ALT: alanine transaminase

Test	Admission	Discharge	Reference range
WBC (L)	8.2	2.8	4.5 to 11.0 × 10^9^/L
Hgb (g/dL)	11.5	9	12-16
Hct (%)	37.3	28.7	41% to 50% for female
MCV (L)	70.6	69.9	80-95
RDW (%)	21.4	21.6	11.5-14.5
Plt count (x10^3^/uL)	616	397	147-425
Calcium (mg/dL)	8	6.8	8.7-10.4
ALT (U/L)	36	27	4-36
Troponin I high Sens (pg/mL)	37.3		< 75 pg/mL for females
Total protein (g/dL)	6.4	5	6-8.3

## Discussion

In this report, we describe the case of a patient with SLE who arrived with pericardial and pleural effusions and was ultimately found to have a COVID-19 infection that led to cardiac tamponade. Her CT scan showed pleural and pericardial effusions and her echocardiography confirmed the clinical diagnosis of cardiac tamponade. She subsequently improved during both hospital visits after undergoing pericardiocentesis and thoracentesis.

In the present study, pericardial and pleural effusions were strongly predicted by the presence of an expanded cardiac profile and a left-sided infiltration on chest radiography (Figure 1) and have previously been reported with COVID-19 infection and SLE. Our findings are well-supported by meta-analysis, which concluded that 4.55% of individuals with suspected or confirmed COVID-19 infection have signs of pericardial effusion on chest CT scans [11].

However, pericardial effusion, pleural effusion, and lymph node enlargement were more common in critically ill patients with COVID-19 pneumonia than in healthy controls [12]. There is still no consensus on what causes pericardial effusion and pleural effusion in COVID-19; however, two main pathways have been proposed [13]. First, the direct binding of the severe acute respiratory syndrome coronavirus 2 (SARS-CoV-2) S protein to human angiotensin-converting enzyme 2 (ACE2) in the human heart may explain the virus's cardiac attraction. Pericardial effusion may develop in a delayed fashion, anywhere from seven days to one month after the onset of symptoms, due to viral replication and subsequent blood circulation. Direct myopericardial lesion by inflammatory cell infiltration is also a reasonable theory that has been documented in COVID-19 cases [14].

More than half of SLE patients display some degree of thoracic involvement, which is more common in SLE than in any other connective tissue disease [15]. In a single-center retrospective study spanning 10 years, researchers analyzed data from 31 people aged <18 years who were diagnosed with SLE. Cardiac involvement was found in 13 people (42%), including two patients with cardiac tamponade [16]. Additionally, another cross-sectional multi-center study of 155 SLE patients (16 years) found that cardiac tamponade was the presenting symptom in two cases (1.3%) [16]. Moreover, 1% of patients with adult-onset SLE had the same presenting symptom in another study [16]. These findings also support the hypothesis that pleural and pericardial effusions can occur in a patient with SLE, which can be possible in our case. Similarly, up to 50% of people with SLE can develop pericardial effusion. Cardiac tamponade due to SLE-caused pericardial effusion is often moderate and asymptomatic and is usually discovered on echocardiography conducted for another reason [17].

Meanwhile, two of the following four symptoms are required to diagnose acute pericarditis: chest pain, a pericardial rub, saddle-shaped ST elevation and/or PR depression, and a nontrivial new or worsening pericardial effusion [17]. Our patient had two out of the four symptoms, including chest discomfort and epicardial and pericardial effusion. The question that can arise is what the mechanism is for the development of pericardial effusion. One possible explanation is that in COVID-19 individuals, pericardial effusion most likely results from a secondary inflammatory response throughout the body. Pericardial inflammation is facilitated by elevated levels of IL-1 and tumor necrosis factor-alpha (TNF-alpha) in the inflammatory cascade [15]. The presence of ACE2 in the heart, cardiomyocytes, and epicardial adipose tissue near the visceral pericardium may further accelerate the development of pericarditis and myopericarditis as direct viral damage [17]. Some scientists have found evidence of the virus in the pericardial effusion by detecting COVID-19 in the pericardial fluid [4]. Severe cases of COVID-19 are associated with hypoxia, which can cause pulmonary hypertension and subsequent pericardial effusion [18]. In the present case study, prior to pericardiocentesis, the patient needed high levels of oxygen, which can be explained by the fact that the heart was pressed upon by the pericardium due to an abnormal accumulation of fluid. Due to stress on the heart, blood was unable to adequately fill the heart's chambers. A cardiac tamponade reduces blood flow and oxygen to the body, which is why the oxygen was supplied.

After two weeks, there was a recurrence of pericardial effusion (Figure 4). Recurrence may be due to a wide variety of infectious and non-infectious triggers that can cause an inflammatory cascade resulting in a recurrence of symptoms. The decrease in WBC count at discharge was also an interesting finding to note. Was the decrease due to the resolution of a combined inflammatory reaction? If SLE caused a severe immunodeficiency, maybe the patient’s baseline WBC count was low to begin with and the sign of a combined effect of COVID-19 and SLE could be tracked by normal WBC measurements in immunodeficient patients and leukopenia after resolution.

## Conclusions

There is currently a dearth of high-quality evidence and guidelines for the management of COVID-19-related pericardial illness or pleural effusion, making it difficult for providers to provide optimal care for their patients. In the present case study, the patient was treated with colchicine due to its well-known effects in treating cardiac tamponade. We found this to be effective in decreasing symptoms of chest pain with the pericardial effusion. While pleural and pericardial effusion have been suggested as complications of coronavirus disease as well as SLE, there is currently insufficient data to support this hypothesis without doing an echocardiogram on all patients with COVID-19 viral infections. The mechanisms behind the cardiac tamponade phenomenon are still not fully understood or able to be defined. In patients displaying a classic "inflammatory phenotype," an auto-inflammatory response may be postulated as a possible mechanism. We do understand that COVID-19 does increase inflammatory response due to IL-1 and TNF-alpha induction. The recurrence of pericardial and pleural effusion with an absent positive COVID-19 test could suggest that SLE had a major contributory role in increasing the occurrence of pericardial effusion. Due to the correlative timing of COVID-19 infection and the occurrence of effusion, the likelihood of a combined effect cannot be eliminated.

We need to find and analyze additional examples to confirm or deny this hypothesis. We hypothesize that there may be other unreported cases of COVID-19-related viral pericarditis, pericardial effusion, and pericardial tamponade, which may be due to the lack of clarity surrounding typical COVID-19 manifestations. Therefore, it is crucial to document and analyze each case of this unique illness. Moreover, based on these results, we recommend that providers of patients with autoimmune diseases advise their patients to look out for symptoms of chest pain with COVID-19 infection, to mitigate potentially extended hospital stays and further complications. In many instances, due to the potential combined effect of COVID-19 and autoimmune disorders, it may be beneficial to screen high-risk patients with an echocardiogram until further research has been done to evaluate the autoimmune interactions.
